# Type I IFNs Are Required to Promote Central Nervous System Immune Surveillance through the Recruitment of Inflammatory Monocytes upon Systemic Inflammation

**DOI:** 10.3389/fimmu.2017.01666

**Published:** 2017-12-04

**Authors:** Javier María Peralta Ramos, Claudio Bussi, Emilia Andrea Gaviglio, Daniela Soledad Arroyo, Natalia Soledad Baez, Maria Cecilia Rodriguez-Galan, Pablo Iribarren

**Affiliations:** ^1^Centro de Investigación en Bioquímica Clínica e Inmunología (CIBICI-CONICET), Departamento de Bioquímica Clínica, Facultad de Ciencias Químicas, Universidad Nacional de Córdoba, Córdoba, Argentina

**Keywords:** microglia, inflammatory monocytes, inflammation, lipopolysaccharide, interferons

## Abstract

Brain-resident microglia and peripheral migratory leukocytes play essential roles in shaping the immune response in the central nervous system. These cells activate and migrate in response to chemokines produced during active immune responses and may contribute to the progression of neuroinflammation. Herein, we addressed the participation of type I–II interferons in the response displayed by microglia and inflammatory monocytes to comprehend the contribution of these cytokines in the establishment and development of a neuroinflammatory process. Following systemic lipopolysaccharide (LPS) challenge, we found glial reactivity and an active recruitment of CD45^hi^ leukocytes close to CD31^+^ vascular endothelial cells in circumventricular organs. Isolated CD11b^+^ CD45^hi^ Ly6C^hi^ Ly6G^−^-primed inflammatory monocytes were able to induce T cell proliferation, unlike CD11b^+^ CD45^lo^ microglia. Moreover, *ex vivo* re-stimulation with LPS exhibited an enhancement of T cell proliferative response promoted by inflammatory monocytes. These myeloid cells also proved to be recruited in a type I interferon-dependent fashion as opposed to neutrophils, unveiling a role of these cytokines in their trafficking. Together, our results compares the phenotypic and functional features between tissue-resident vs peripheral recruited cells in an inflamed microenvironment, identifying inflammatory monocytes as key sentinels in a LPS-induced murine model of neuroinflammation.

## Introduction

Immunological surveillance of the central nervous system (CNS) is dynamic, specific, and tightly regulated. During neuroinflammation, the blood–brain barrier (BBB) might get disrupted, enabling peripheral immune cells to gain access to the brain parenchyma. Brain-resident microglia encounter myeloid immune cells that have been primed in the periphery, establishing an interplay that could lead to the development and worsening of this inflammatory process with a detrimental outcome (1, 2).

Systemic injection of the endotoxin lipopolysaccharide (LPS), a cell wall component of Gram-negative bacteria and a canonical ligand for toll-like receptor 4 (TLR4), has been widely used as an inflammatory model (3–14). These peripherally applied stimuli leads to a cytokine-storm that signals to the brain, inducing a decline in BBB integrity and triggering an immune response within it.

Tissue-resident microglia represent the first line of defense against invading pathogens and modulate neuroinflammation. Despite being extremely long-lived, microglia exhibit considerable self-renewal (15) and are highly active surveillants of the CNS under steady-state conditions (16). Peripheral blood-derived monocytes consist of two functional subsets, a patrolling CX_3_CR1^hi^ CCR2^−^ subset and an inflammatory CX_3_CR1^lo^ CCR2^+^ subset (17) with the ability to migrate to inflamed tissues and differentiate into dendritic cells (18) or microglia under defined conditions (19, 20).

Current knowledge in neuroimmunology remains scarce. The recognition of a lymphatic drainage system of the CNS has drawn attention to the meninges and the choroid plexus, challenging the established basic assumptions of the CNS immune privilege (21, 22). In this regard, type I and II interferon (IFN-a/b and IFN-g) pathways have proved to be part of an elaborate cytokine network regulating the migration of immune cells through these CNS gateways (23), seemingly yielding opposite effects.

There is growing evidence that systemic inflammatory events can have devastating effects in the brain (4, 24, 25) due to their impact in the progression of several CNS disorders (11, 26), such as autoimmune and neurodegenerative diseases in which leukocyte recruitment is a key feature. Understanding the impact of systemic inflammation in myeloid cell trafficking into the CNS and the underlying mechanisms involved in this process is determining.

In our study, we shed light on the differential roles of microglia and inflammatory monocytes (27, 28) and contributed to elucidate the participation of type I–II interferons during the immune response elicited by these tissue-resident versus peripheral recruited cells in a lipopolysaccharide (LPS)-induced murine model of neuroinflammation.

## Materials and Methods

### Animals and LPS Administration

Wild-type (WT) C57BL/6 mice were originally obtained from School of Veterinary, National University of La Plata. IFN-g^−/−^ (B6.129S7-Ifngtm1Ts/J strain) mice were obtained from The Jackson Laboratory and IFNAR^−/−^ (Ifnar1tm1Ag strain) mice were kindly provided by Institut Pasteur. Between 8- and 12-week-old male mice were maintained in the specific pathogen-free barrier facilities at the School of Chemical Sciences animal facility, where all experiments were done in compliance with the procedures outlined in the “Guide for the Care and Use of Laboratory Animals” (NIH Publication No. 86-23, 1985). The experimental protocols were approved by the Institutional Animal Care and Use Committee (IACUC). Our animal facility obtained NIH animal welfare assurance (No. A5802-01, OLAW, NIH, US).

Lipopolysaccharide from *Escherichia coli* 055:B5 (purified by gel-filtration chromatography) was purchased from Sigma-Aldrich and freshly dissolved in sterile saline prior to intraperitoneal (i.p.) injection. Mice were treated with either vehicle or 40 µg of LPS (1,6 mg/kg) for four consecutive days to induce neuroinflammation, following an injection scheme modified from Cardona et al. (5).

### Isolation of Immune Cells from Mice Brains

Twelve hours post the last i.p. injection, mice were weighed and deeply anesthetized with a ketamine/xylazine cocktail according to their weight. Immune cells were isolated from whole brain homogenates as follows. Briefly, mice were transcardially perfused with ice-cold PBS (Gibco), and brains were collected in DMEM (Gibco) supplemented with sodium pyruvate (Gibco) and a penicillin, streptomycin, and glutamine cocktail (Gibco), gently disaggregated mechanically and resuspended in PBS containing 3 mg/mL collagenase D (Roche Diagnostics) plus 10 µg/mL DNAse (Sigma-Aldrich) for an enzymatically homogenization. After this incubation, brain homogenates were filtered in 40-µm pore size cell strainers (BD Biosciences), centrifuged 8 min at 1,800 rpm, washed with PBS, and resuspended in 6 mL of 38% isotonic Percoll^®^ (GE Healthcare) before a 25-min centrifugation at 800*g* without neither acceleration nor brake. Myelin and debris were discarded. Cell pellets containing total brain immune cells were collected, washed with DMEM supplemented with 10% fetal bovine serum (Gibco), and cell viability was determined by trypan blue exclusion using a Neubauer’s chamber. Finally, cells were labeled for subsequent flow cytometric analysis or cell sorting.

### Flow Cytometric Analysis and Cell Sorting

Surface staining of single-cell suspension of isolated brain immune cells was performed using standard protocols and analyzed on a FACS Canto II (BD Biosciences) or sorted on a FACS Aria III (BD Biosciences). Sort gates were defined based on the expression of CD11b, CD45, Ly6C, and Ly6G as follows: microglial cells, CD11b^+^ CD45^lo^; neutrophils, CD11b^+^ CD45^hi^ Ly6C^+^ Ly6G^+^; inflammatory monocytes, CD11b^+^ CD45^hi^ Ly6C^hi^ Ly6G^−^. Data analysis was conducted using FCS express (*De Novo* Software). The following antibodies were used in the procedure: monoclonal anti-mouse CD11b APC (BioLegend, clone M1/70), CD11b FITC (BD Pharmingen, clone M1/70), CD45 APC-Cy7 (BioLegend, clone 30-F11), CD11c PerCP (BD Pharmingen, clone N418), Ly6C PE-Cy7 (BD Pharmingen, clone AL-21), Ly6G PE (BD Pharmingen, clone 1A8), I-A/I-E Alexa Fluor 647 (BioLegend, clone M5/114.15.2), FcϵRI PE-Cy7 (eBioscience, clone MAR-1), CCR2 (Abcam, clone E68), or polyclonal anti-mouse CX_3_CR1 (Abcam) plus Alexa Fluor 488 (Molecular Probes) antibody or isotype control antibodies (BD Pharmingen, APC, clone R35-95; PerCP/PE, clone A95-1; PE-Cy7, clone G155-178). The assessment of intracellular expression of chemokine receptors was performed according to the Cytofix/Cytoperm™ fixation/permeabilization solution kit (BD Biosciences) manufacturer’s instructions. Briefly, cells were surface-labeled as mentioned above. Then, samples were fixed and permeabilized for 20 min at 4°C with Fixation/Permeabilization solution and washed with BD Perm/Wash buffer™. Next, cells were incubated with BD Perm/Wash buffer™ containing monoclonal anti-mouse CCR2 (Abcam, clone E68) or polyclonal anti-mouse CX_3_CR1 (Abcam). Finally, samples were washed with BD Perm/Wash buffer™ and resuspended in the same buffer containing Alexa Fluor 488 antibody (Molecular Probes).

### *Ex Vivo* Suppression Assays

Microglial cells or inflammatory monocytes isolated from endotoxemic mice, stimulated or not with a LPS (100 ng/mL) plus interferon gamma (IFN-g, 20 ng/mL) (Peprotech) cocktail were cocultured with splenocytes derived from naïve control mice and previously stained with CFSE (4 µM) (Molecular Probes), at a 1:1 ratio (1 × 10^5^ cell/mL). For mitogenic-induced cell proliferation, cocultures were maintained for 72 h in round-bottom 96-well plates in the presence or absence of Concanavalin A (Con A, 5 µg/mL) (Sigma-Aldrich) in RPMI (Gibco) supplemented with 10% fetal bovine serum (Gibco). Cells were then harvested and stained with CD4 APC (BD Pharmingen, clone RM4-5) and CD8 PE (BD Pharmingen, clone 53-6.7) and analyzed as mentioned above.

### Reverse Transcription of mRNA and Quantification by Real-time PCR

Brain homogenates or isolated cells were incubated with TRIzol^®^ (Invitrogen), then RNA was extracted according to the manufacturer’s instructions and stored at −80°C. Total RNA was quantified using a Synergy HT spectrophotometer (BioTek) and 1 µg was treated with DNAse (Sigma-Aldrich) and reverse-transcribed using the High-Capacity cDNA Reverse Transcription Kit (Applied Biosystems) and following the manufacturer’s protocol. Real-time PCR was performed on a StepOnePlus™ real-time PCR system (Applied Biosystems) using SYBR^®^ Green real-time PCR master mix (Applied Biosystems), and relative quantification (RQ) was calculated by using StepOne™ software V2.2.2, based on the equation RQ = 2^−ΔΔCt^, where Ct is the threshold cycle to detect fluorescence. Ct data were normalized to the internal standard HPRT1. Primer sequences were as follows: CCL2, sense: CCC ACT CAC CTG CTA CT, anti-sense: TCT GGA CCC ATT CCT TCT TG; CCR2, sense: GTG TGA TTG ACA AGC ACT TAG ACC, anti-sense: GGA GAG ATA CCT TCG GAA CTT CTC; CX_3_CL1, sense: CGA AAT GCG AAA TCA TGT GCG AC, anti-sense: GAC TCC TGG TTT AGC TGA TAG CG; CX_3_CR1: GGA CTC ACT ACC TCA GCC, anti-sense: TCC GGT TGT TCA TGG AGT TGG; CXCL1, sense: CAC CTC AAG AAC ATC CAG AGC, anti-sense: GGT CGC GAG GCT TGC CTT GA; CXCR2, sense: CTG GCA TGC CCT CTA TTC TGC, anti-sense: GCT GGT CAT CTT ATA CAA CGG G; IFN-b, sense: TTA CAC TGC CTT TGC CAT CC, anti-sense: ACT GTC TGC TGG AGT TCA T; IFNAR, sense: CGA GGC GAA GTG GTT AAA A, anti-sense: ACG GAT CAA CCT CAT TCC AC; HPRT1, sense: TCA GTC AAC GGG GGA CAT AAA, anti-sense: GGG GCT GTA CTG CTT AAC CAG.

### H&E Staining and Immunofluorescence

Twelve hours post the last i.p. injection, mice were weighed and deeply anesthetized with a ketamine/xylazine cocktail according to their weight. Animals were transcardially perfused once with ice-cold PBS (Gibco) and then with 4% paraformaldehyde. Brains were collected in 4% paraformaldehyde for an additional 24 h post-fixation and incubated in 20% sucrose for 24 h more.

For H&E staining, brains were embedded in paraffin, cut into 10 µm sections of thickness using a Shandon Cryotome E cryostat (Thermo Scientific), and mounted on Starfrost^®^ adhesive slides (Knittel Glass). Then, sections were immersed in hematoxylin and rinsed in distilled water. Next, slides were immersed in eosin and rinsed three times with 95% isopropilic alcohol. Following that, sections were quickly rinsed in a combined solution of xylene and ethanol and twice in xylene. Finally, slides were mounted with a drop of Canada balsam and analyzed under an Eclipse TE 2000-Ulight microscope (Nikon) with an ACT-2U digital camera (Nikon) attached to it to capture the images.

For immunofluorescence, brains were embedded in Tissue-Tek^®^ optimal cutting temperature compound (Sakura), cut into 10 µm sections of thickness using a Shandon Cryotome E cryostat (Thermo Scientific), and mounted on Starfrost^®^ adhesive slides (Knittel Glass). Sections were rehydrated with blocking buffer (10% BSA, 0.3% Triton in TBS), rinsed with TBS (Gibco), and incubated overnight at 4°C with the corresponding dilutions of the antibodies CD45 (BioLegend, clone 30-F11), CD31 (Santa Cruz, clone M-20), or glial fibrillary acidic protein (Abcam, clone GF5) in blocking buffer. After several rinses, sections were incubated with Alexa Fluor 488 (Molecular Probes), Alexa Fluor 546 (Molecular Probes), or Alexa Fluor 633 (Molecular Probes) antibodies and counterstained with DAPI. Slides were analyzed under a FV1000 laser scanning confocal fluorescence microscope (Olympus).

### Statistical Analysis

Results are expressed as mean ± SEM. Data distribution was assumed to be normal, but this was not formally tested for all experiments. All statistical analyses were performed using Prism^®^ 7.0 (GraphPad software). Means between two groups were compared with unpaired *t*-test. Means between three or more groups were compared with two-way analysis of variance followed by a Tuckey’s *post hoc* test. Statistical significance levels were set as follows: * if *p* < 0.05, ** if *p* < 0.01, and *** if *p* < 0.001.

## Results

### Systemic LPS Challenge Induces Glial Activation and Recruitment of Peripheral CD11b^+^ CD45^hi^ Ly6C^hi^ Ly6G^−^ Cells into the CNS

To assess neuroinflammation and to establish the cellular players involved in this process after a systemic LPS challenge, we evaluated glial activation and the recruitment of peripheral immune cells to the CNS through a flow cytometry multiparametric gating analysis strategy (Figure S1 in Supplementary Material).

**Figure 1:**
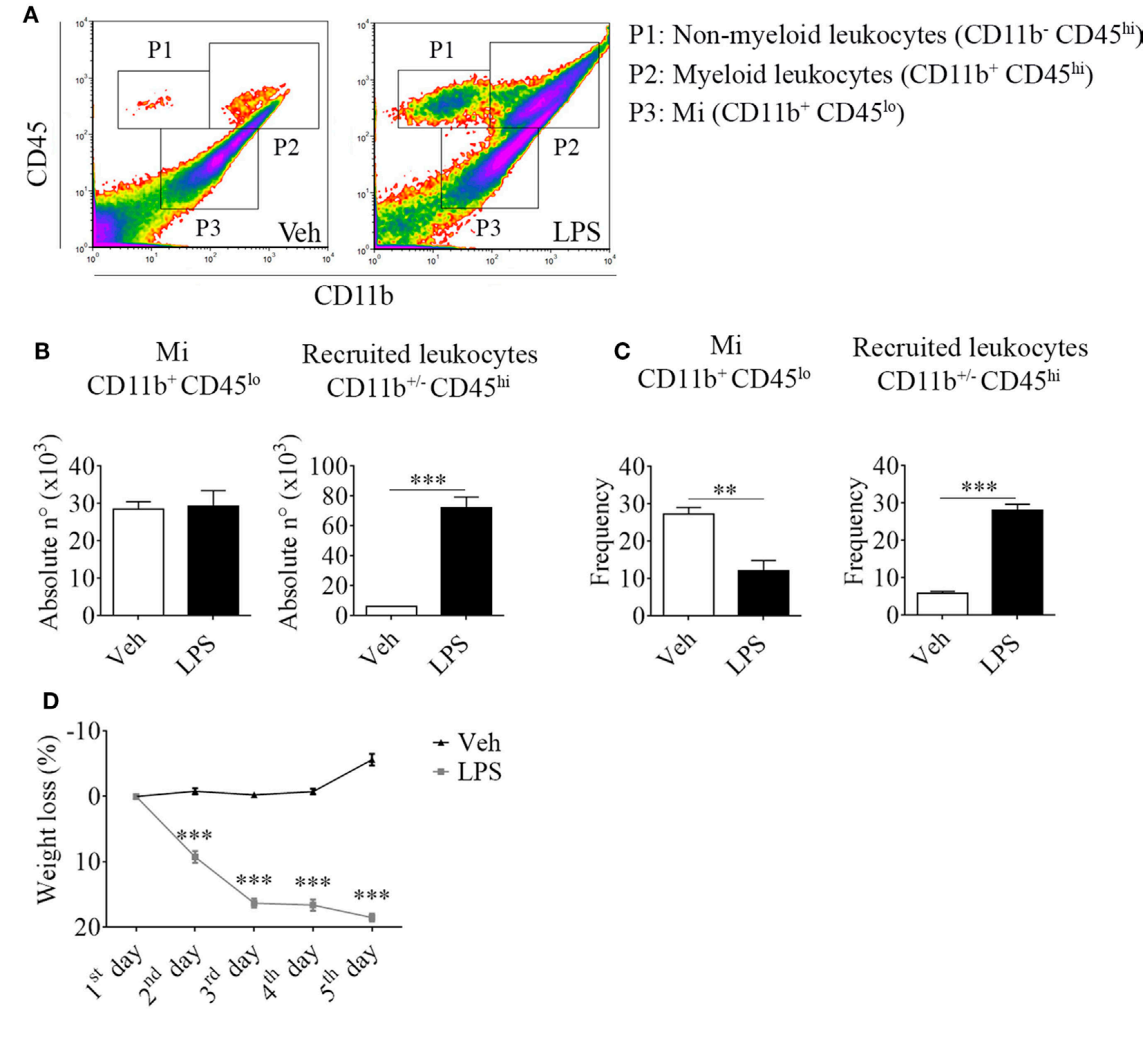
Lipopolysaccharide (LPS) stimulation induces the recruitment of peripheral CD11b^+/−^ CD45^hi^ cells to the central nervous system. Mice were treated i.p. with either vehicle or 40 µg of LPS (1,6 mg/kg) for four consecutive days to induce neuroinflammation. Twelve hours post the last injection, mice were euthanized and immune cells were isolated from whole brain homogenates and labeled for subsequent flow cytometric analysis. **(A)** Representative CD45 vs CD11b flow cytometry density-plots illustrating the gating analysis strategy employed. P1: CD11b^−^ CD45^hi^ lymphoid recruited leukocytes; P2: CD11b^+^ CD45^hi^ myeloid recruited leukocytes; P3: CD11b^+^ CD45^lo^ microglial cells. **(B)** Absolute number and **(C)** frequency of CD11b^+^ CD45^lo^ microglial cells, CD11b^+/−^ CD45^hi^ recruited leukocytes, CD11b^+^ CD45^hi^ Ly6C^+^ Ly6G^+^ neutrophils, and CD11b^+^ CD45^hi^ Ly6C^hi^ Ly6G^−^ inflammatory monocytes, were assessed by flow cytometry. The percentage of these two last populations corresponding when gated in CD11b^+^ CD45^hi^. Results are representative of at least three independent experiments (*n* = 3–4 animals per group). **(D)** Mice weight loss following the i.p. administration scheme with vehicle or 40 µg of LPS (1.6 mg/kg) for four consecutive days to induce neuroinflammation. Results are representative of at least three independent experiments (*n* = 3–20 animals per group). Data are expressed as mean ± SEM. Mi, microglial cells; PMN, neutrophils; Inf Mo, inflammatory monocytes.

**Figure 2:**
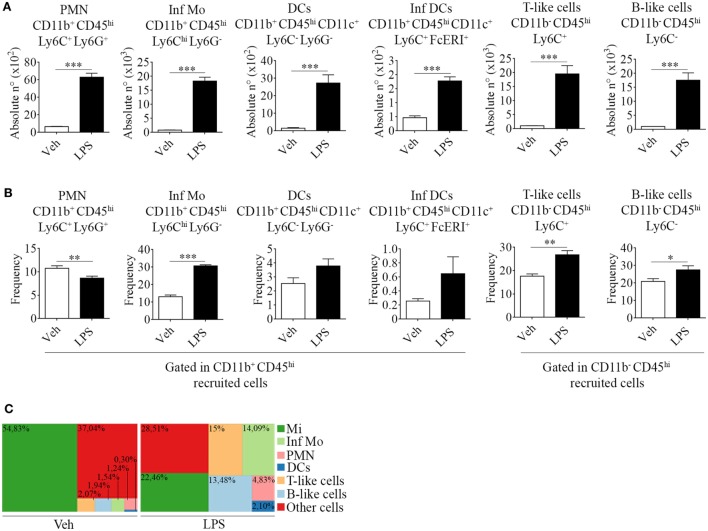
Phenotypic features of immune cells involved in neuroinflammation in the brain from endotoxemic mice. Mice were treated i.p. with either vehicle or 40 µg of lipopolysaccharide (LPS) (1,6 mg/kg) for four consecutive days to induce neuroinflammation. Twelve hours post the last injection, mice were euthanized and immune cells were isolated from whole brain homogenates and labeled for subsequent flow cytometric analysis. **(A)** Absolute number and **(B)** frequency of CD11b^+^ CD45^hi^ CD11c^+^ Ly6C^−^ Ly6G^−^ dendritic cells, CD11b^+^ CD45^hi^ CD11c^+^ Ly6C^+^ FcϵRI^+^ inflammatory dendritic cells, CD11b^−^ CD45^hi^ Ly6C^+^ T-like, and CD11b^+^ CD45^hi^ Ly6C^−^ B-like lymphocytes, was assessed by flow cytometry. The percentages shown correspond to populations gated as CD11b^+^ CD45^hi^. Results are representative of at least two independent experiments (*n* = 3–4 animals per group). **(C)** Representative hierarchical tree map comparing vehicle vs LPS frequency of resident and peripheral populations previously analyzed by flow cytometry. Results are representative of at least three independent experiments (*n* = 3–4 animals per group). Data are expressed as mean ± SEM. Mi, microglial cells; Inf Mo, inflammatory monocytes; PMN, neutrophils; DCs, dendritic cells; Inf DCs, inflammatory dendritic cells; other cells, neurons, astrocytes, oligodendrocytes.

For this purpose, we took advantage of the differential expression of the myeloid surface antigen CD11b and the pan-leukocyte marker CD45 in tissue-resident microglial cells (CD11b^+^ CD45^lo^) and peripheral recruited immune cells (CD11b^+/−^ CD45^hi^) (Figures 1A–C). Following LPS stimulation and based on these selection criteria, we found no changes in the absolute number but a decrease in the frequency of microglial cells due to the overwhelming recruitment of peripheral leukocytes. This effect upon LPS injection was accompanied by a marked weight loss, probably due to the development of sickness behavior (Figure 1D). To characterize the phenotypic features of the leukocyte trafficking to the CNS, we identified neutrophils (CD11b^+^ CD45^hi^ Ly6C^+^ Ly6G^+^) and inflammatory monocytes (CD11b^+^ CD45^hi^ Ly6C^hi^ Ly6G^−^) as the major population of CNS-associated phagocytes in LPS-treated mice (Figures 2A,B) among innate myeloid CD11b^+^ CD45^hi^ leukocytes.

Correspondingly, we noticed an increase in the recruitment of professional antigen presenting dendritic cells (CD11b^+^ CD45^hi^ CD11c^+^ Ly6C^−^ Ly6G^−^) and of a distinctive subset, derived from monocytes that differentiate *in situ* under inflammatory circumstances, termed inflammatory dendritic cells (CD11b^+^ CD45^hi^ CD11c^+^ Ly6C^+^ FcϵRI^+^) (Figures 2A,B). Furthermore, we found CD11b^−^ CD45^hi^ T (Ly6C^+^) and B-like (Ly6C^−^) cells (Figures 2A,B). Our results clearly show that an inflammogen, such as LPS, promotes the redistribution of the frequencies of different populations of leukocytes recruited to the CNS when compared to those of neuronal or glial populations (Figure 2C).

**Figure 3:**
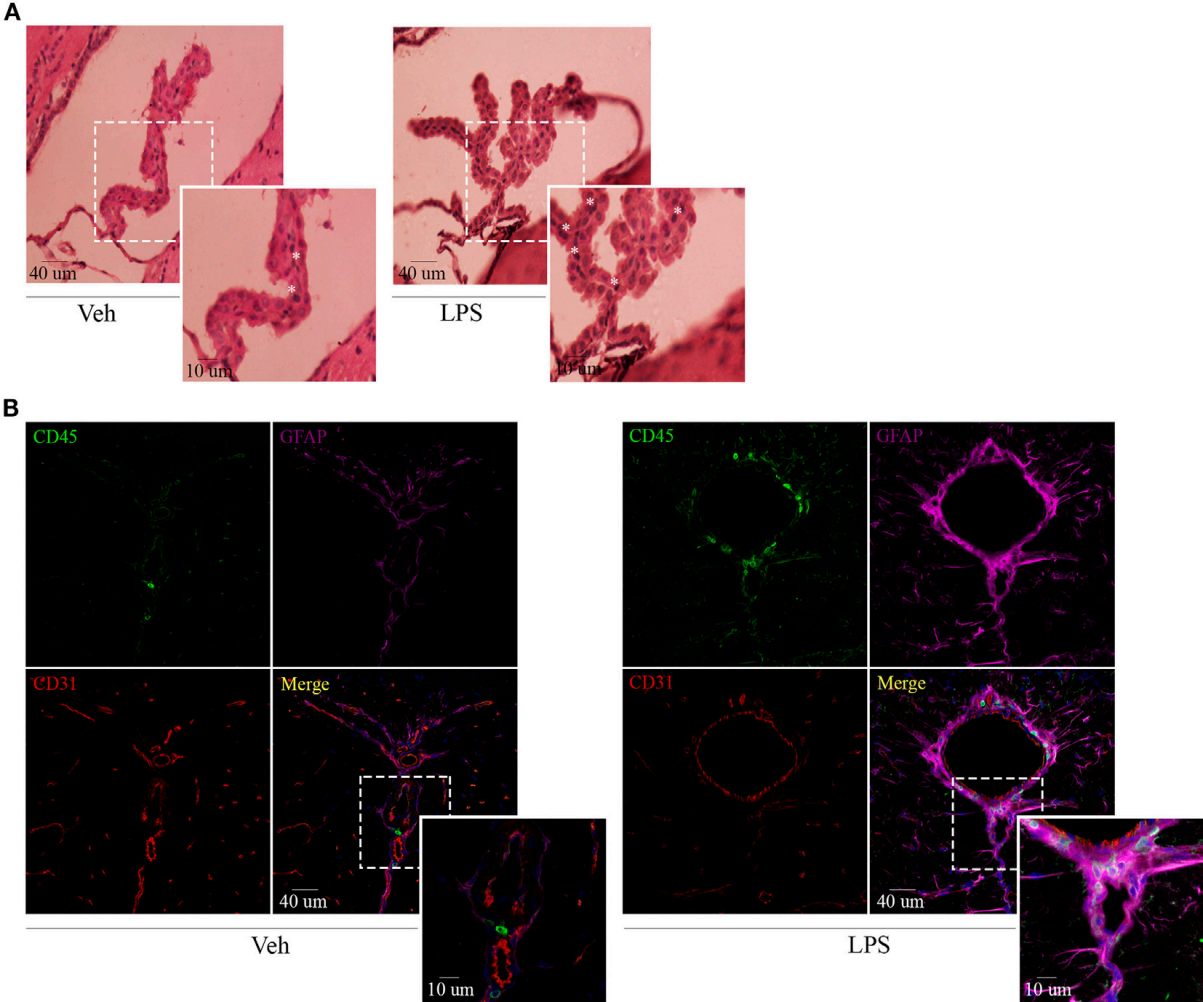
Lipopolysaccharide (LPS) challenge induces glial activation and recruitment of leukocytes to CVOs. Mice were treated i.p. with either vehicle or 40 µg of LPS (1,6 mg/kg) for four consecutive days to induce neuroinflammation. Twelve hours post the last injection, mice were euthanized and brains collected. **(A)** H&E micrographs of the choroid plexus. Scale bars, 40 µm (main panels), 13 µm (inset). The asterisks denote recruited leukocytes. Results are representative of at least two independent experiments (*n* = 3–4 animals per group). **(B)** Confocal micrographs of CVOs depicting cluster of differentiation (CD45)-low microglial cells and high recruited leukocytes (upper-left, green), glial fibrillary acidic protein-positive astrocytes (upper-right, false-colored magenta), and cluster of differentiation (CD31)-positive endothelial cells (lower-left, red). DAPI counterstain (blue) shows nucleus. Scale bars, 40 µm (main panels), 13 µm (inset). Results are representative of at least three independent experiments (*n* = 3–4 animals per group).

Recent findings have revealed a role for circumventricular organs as gateways in the trafficking of peripheral leukocytes to the CNS. By virtue of this, we next assessed neuroinflammation and determined the location of the leukocytes recruited to the brain by both immunohistochemistry and immunofluorescence. Hematoxylin and eosin staining revealed recruitment of leukocytes in choroid plexus of endotoxemic mice (Figure 3A), confirming the involvement of this site in the immune surveillance of the brain. As mentioned above, when using confocal microscopy, we exploited both the morphology and the mild expression of the cluster of differentiation CD45 in star-shaped brain-resident microglial cells to distinguish them from rounded peripheral leukocytes bearing a high expression of this marker. Thus, systemic LPS induced microglial and astrocytic reactivity as shown by the shrinkage and thickening of glial processes and promoted the recruitment of inflammatory leukocytes, particularly close to vascular CD31-expressing endothelial cells in circumventricular organs (Figure 3B). Our results exhibited no infiltration of immune cells into the brain parenchyma but remain in the perivascular compartment, suggesting that at least under these circumstances, there may be no signals in the microenvironment to do so.

### LPS Stimulation Modulates CCR2 and CX_3_CR1 Expression in Tissue-Resident CD11b^+^ CD45^lo^ and Peripheral CD11b^+^ CD45^hi^ Ly6C^hi^ Ly6G^−^ Cells

Chemokines and their receptors are active players inducing the access of myeloid cells to the CNS. To better understand microglial activation and the selective migration of inflammatory monocytes following a LPS challenge, we next assessed the status of CCL2/CCR2 and CX_3_CL1/CX_3_CR1 axes.

**Figure 4:**
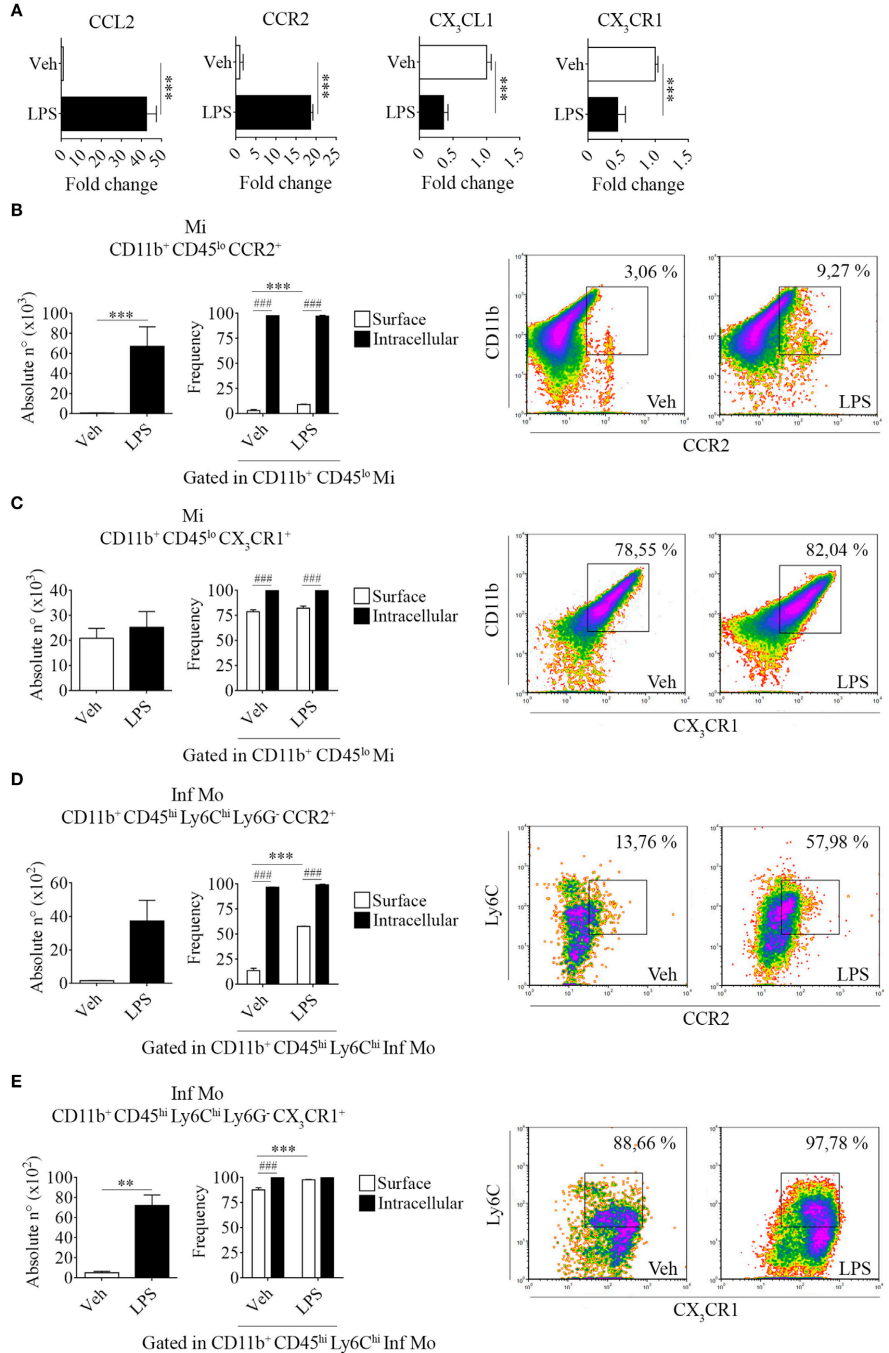
Increase of brain-resident CD11b^+^ CD45^lo^ and peripheral CD11b^+^ CD45^hi^ Ly6C^hi^ Ly6G^−^ cells bearing CCR2 and CX_3_CR1 in lipopolysaccharide (LPS)-treated mice. Mice were treated i.p. with either vehicle or 40 µg of LPS (1,6 mg/kg) for four consecutive days to induce neuroinflammation. Twelve hours post the last injection, mice were euthanized and RNA extracted from whole brain homogenates cells or immune cells isolated and labeled for subsequent flow cytometric analysis. **(A)** Gene expression analysis by real-time PCR of CCL2, CX_3_CL1 and CCR2, CX_3_CR1 in total bulk brain. Results are representative of at least two independent experiments (*n* = 3 animals per group). Relative quantification (RQ) was calculated based on the equation RQ = 2^−ΔΔCt^, where Ct is the threshold cycle to detect fluorescence. Ct data were normalized to the internal standard HPRT1. **(B,D)** Absolute number and **(C,E)** frequency of CD11b^+^ CD45^lo^ microglial cells and CD11b^+^ CD45^hi^ Ly6C^hi^ Ly6G^−^ inflammatory monocytes expressing CCR2 and CX_3_CR1, was assessed by flow cytometry. The percentage of these populations corresponding when gated in CD11b^+^ CD45^lo^ or CD11b^+^ CD45^hi^ Ly6C^hi^, respectively. Representative density-plots illustrate the gating analysis strategy employed. Results are representative of two independent experiments (*n* = 3–6 animals per group). Data are expressed as mean ± SEM. Mi, microglial cells; Inf Mo, inflammatory monocytes.

Systemic LPS conversely modulated these chemokine/receptor axes, as determined by an increase in CCL2 and CCR2 gene expression levels but a decrease in both CX_3_CL1 and CX_3_CR1 in total bulk brains from LPS-treated mice when compared to their vehicle-treated counterparts (Figure 4A). Subsequently, we performed surface and intracellular staining of chemokine receptors CCR2 and CX_3_CR1 in tissue-resident microglia and peripheral inflammatory monocytes to further characterize the response exerted by LPS in these populations. Thereby, we noticed an increase in the absolute number of CCR2 surface-bearing microglia (Figures 4B,C and Figure S2 in Supplementary Material) and CX_3_CR1^+^ inflammatory monocytes (Figures 4D,E and Figure S2 in Supplementary Material) after LPS systemic administration. These results imply a positive feedback between the unhindered recruitment of CCR2^+^ cells and the exacerbation of inflammation through the downregulation of inhibitory CX_3_CL1 and CX_3_CR1, as shown by quantitative real-time PCR. Likewise, flow cytometry assays showed that despite not always promoting the expression of either CCR2 or CX_3_CR1 in the surface of microglia and inflammatory monocytes, LPS upregulates the expression of both chemokine receptors intracellularly in these populations.

### Plasticity of LPS-Primed Inflammatory CD11b^+^ CD45^hi^ Ly6C^hi^ Ly6G^−^ Cells in the Regulation of T Cell Proliferation

To date, several studies have described invading blood-derived monocytes as highly mobile and inflammatory, whereas resident microglia have been characterized as active motile CNS myeloid cells that function as the first line of defense against invading pathogens. Therefore, we addressed the hypothesis that inflammatory monocytes and microglia could be playing different roles in neuroinflammation by either promoting or suppressing T cell proliferative response.

**Figure 5:**
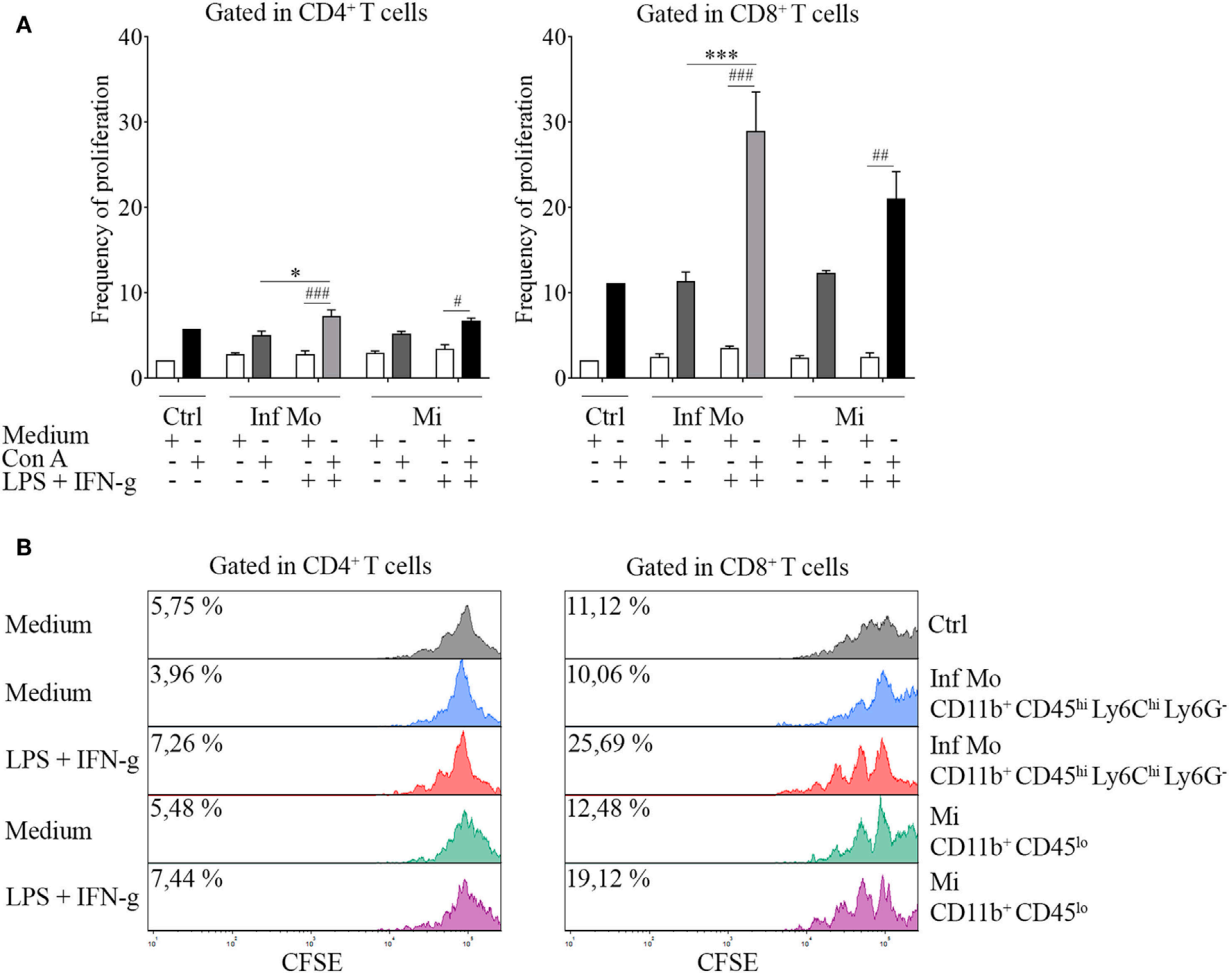
Immune function of inflammatory CD11b^+^ CD45^hi^ Ly6C^hi^ Ly6G^−^ cells isolated after a systemic lipopolysaccharide (LPS) challenge. Mice were treated i.p. with either vehicle or 40 µg of LPS (1,6 mg/kg) for four consecutive days to induce neuroinflammation. Twelve hours post the last injection, mice were euthanized and immune cells were isolated from whole brain homogenates and labeled for subsequent cell sorting. **(A)** *In vivo* LPS-primed CD11b^+^ CD45^hi^ Ly6C^hi^ Ly6G^−^ inflammatory monocytes or CD11b^+^ CD45^lo^ microglial cells isolated by cell sorting from a pool of mice brains were cocultured with CFSE (4 µM)-labeled naïve splenocytes at a 1:1 ratio (1 × 10^5^ cell/mL) in the presence or absence of concanavalin A (Con A) (5 µg/mL). Cells from endotoxemic mice were challenged or not*ex vivo* with LPS (100 ng/mL) plus IFN-g (20 ng/mL) cocktail prior to the coculture. T cell frequency of proliferation was examined after 72 h by CFSE dilution through flow cytometry. Results are representative of two independent experiments combined (*n* = 17–20 animals). **(B)** Representative stacked histograms depicting CFSE dilution. Data are expressed as mean ± SEM. Ctrl, control; Inf Mo, inflammatory monocytes; Mi, microglial cells.

To tackle this issue, we performed a proliferation assay in which CNS isolated inflammatory monocytes or microglia were cocultured with naïve splenocytes in the presence or not of Con A. Interestingly, we found that LPS-primed inflammatory monocytes were able to induce T cell proliferation unlike microglia, in an increasing ratio-dependent manner (data not shown). To gain insight into this mechanism, we sought to test if peripheral isolated inflammatory monocytes and tissue-resident microglia might have functional plasticity by exposing or not these cells to a repeated *ex vivo* LPS challenge in addition with IFN-g as a combination of multiple activation signals prior to the coculture. Surprisingly, boosting of the cells showed an enhancement of T cell proliferation by inflammatory monocytes when compared to the single stimulation condition (Figure 5A), as pictured by the representative CFSE dilution stacked histograms (Figure 5B). These results demonstrate monocytes and microglia are sensitive to pro-inflammatory re-stimulation and could differentially regulate T cell subsets multiplication, as suggested by CD4^+^ T cell weakened proliferative response in comparison with the CD8^+^ T cell proliferation.

### Type I IFNs Are Required to Promote CD11b^+^ CD45^hi^ Ly6C^hi^ Ly6G^−^ Cell Recruitment to the CNS in Endotoxemic Mice

Recent studies proposed IFNs as crucial mediators in brain function but many questions regarding the role of these molecules in the CNS remain unanswered yet. We appealed to our previously described flow cytometric analysis strategy to delve deeper in the understanding of these cytokines and evaluate whether if they could be participating in the modulation of neuroinflammation.

**Figure 6:**
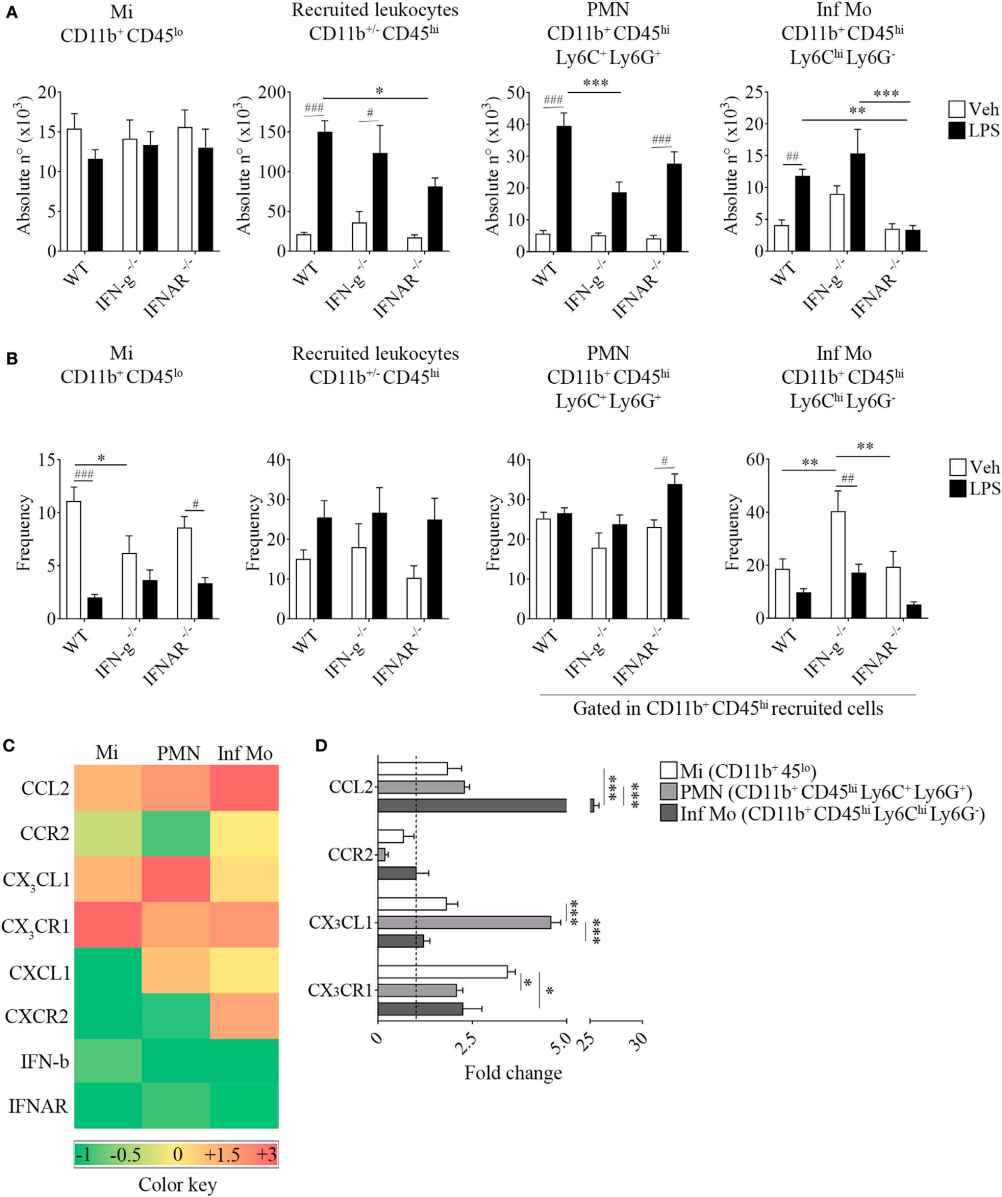
Impaired recruitment of inflammatory CD11b^+^ CD45^hi^ Ly6C^hi^ Ly6G^−^ cells in IFNAR^−/−^ endotoxemic mice. Mice were treated i.p. with either vehicle or 40 µg of lipopolysaccharide (LPS) (1,6 mg/kg) for four consecutive days to induce neuroinflammation. Twelve hours post the last injection, mice were euthanized and immune cells were isolated from whole brain homogenates and labeled for subsequent flow cytometric analysis or cell sorting. **(A)** Absolute number and **(B)** frequency of CD11b^+^ CD45^lo^ microglial cells, CD11b^+/−^ CD45^hi^ recruited leukocytes, CD11b^+^ CD45^hi^ Ly6C^+^ Ly6G^+^ neutrophils, and CD11b^+^ CD45^hi^ Ly6C^hi^ Ly6G^−^ inflammatory monocytes derived from wild-type (WT), IFN-g^−/−^, and IFNAR^−/−^ mice, were assessed by flow cytometry. Results are an average of three independent experiments (*n* = 3–4 animals per group) for each knockout mice strain. **(C)** Heat map rendering the gene expression analysis (fold increase) by real-time PCR of CCL2, CX_3_CL1, CXCL1, IFN-b and CCR2, CX_3_CR1, CXCR2, IFNAR of LPS-primed CD11b^+^ CD45^lo^ microglial cells, CD11b^+^ CD45^hi^ Ly6C^+^ Ly6G^+^ neutrophils and CD11b^+^ CD45^hi^ Ly6C^hi^ Ly6G^−^ inflammatory monocytes isolated from a pool of endotoxemic WT vs IFNAR^−/−^ mice brains. Relative quantification (RQ) was calculated based on the equation RQ = 2^−ΔΔCt^, where Ct is the threshold cycle to detect fluorescence. Ct data were normalized to the internal standard HPRT1. Results are representative of two independent experiments (*n* = 9–17 animals per group). **(D)** Further comparison between CCL2, CX_3_CL1 and CCR2, CX_3_CR1 gene expression of LPS-primed CD11b^+^ CD45^lo^ microglial cells, CD11b^+^ CD45^hi^ Ly6C^+^ Ly6G^+^ neutrophils, and CD11b^+^ CD45^hi^ Ly6C^hi^ Ly6G^−^ inflammatory monocytes from WT (dashed line) vs IFNAR^−/−^ mice. Data are expressed as mean ± SEM. Mi, microglial cells; PMN, neutrophils; Inf Mo, inflammatory monocytes.

Following a systemic challenge with LPS, we did not find any variation in the absolute number of microglia but a decrease in the absolute number of recruited peripheral leukocytes in IFNs deficient mice strains compared to WT mice. This effect upon LPS injection in KO mice was accompanied by a reduced weight loss (Figure S3 in Supplementary Material), probably correlating with the observed decline in the immune cell recruitment to their CNS. Unexpectedly, we found LPS favored the recruitment of peripheral inflammatory monocytes rather than neutrophils in animals devoid of IFN-g. Moreover, inflammatory monocytes proved to be recruited to the CNS in a type I IFN-dependent fashion as opposed to neutrophils, as shown by the impairment in their recruitment in IFN-a/b receptor knockout (IFNAR^−/−^) brains (Figures 6A,B). In this sense, only LPS-challenged WT mice showed an increase in the recruitment of peripheral major histocompatibility class II (MHC II)-expressing inflammatory monocytes (Figure S4 in Supplementary Material). In order to clarify the underlying mechanism whereby peripheral inflammatory monocytes lacking the type I IFN receptor could mildly migrate to the CNS upon a systemic LPS administration, we then set out to identify the molecular mediator involved in this response. Thus, we separately isolated microglia, neutrophils and inflammatory monocytes and assessed the gene expression of several chemokine/receptor axes considered critical for the trafficking of these cells to the brain. As depicted by the representative gene array chart (Figure 6C), the increase in CCL2 gene expression levels in inflammatory monocytes suggest a redundant function for the recruitment of these cells. Indeed, the upregulation of CX_3_CL1 in neutrophils (Figures 6C,D) implies a crosstalk of this chemokine with type I IFNs as well as a requirement of both mediators for a proper immune cell entry to the CNS under inflammatory circumstances.

## Discussion

The results presented identify inflammatory monocytes as gatekeepers of CNS immune surveillance (29) upon a LPS peripheral challenge, as determined by the augmentation of their recruitment when compared to other immune cell populations. This is in agreement with Cazareth et al. (12), which shows an enhanced migration of inflammatory monocytes but neither trafficking of neutrophils nor T and B lymphocytes to the CNS by means of a similar flow cytometry approach, following a single and acute systemic administration of LPS. On the contrary, the finding of a consistent recruitment of these latter cells in our experimental settings demonstrates that the outcome of a proposed model varies depending on the dose and timing regime of LPS exposure. Several studies have described the design of LPS models to assess the implications of central or peripheral inflammation in the development of a less-common neurodegenerative condition such as prion disease (4, 11, 26) or even to induce and mimic features of Alzheimer’s (8) and Parkinson’s disease (7) in naïve animals. Noteworthy, Ruiz-Valdepenas et al. (9) showed by intravital microscopy increased extravasation of dextran after a single moderate dose of LPS so it is certainly likely that in our neuroinflammation model this inflammogen would be gaining access, via BBB breakdown, to the parenchyma and activating directly to brain cells.

Similar to previous reports during pathogenic inflammation in experimental autoimmune encephalomyelitis (30, 31), we determined the presence of a peculiar subset of dendritic cells, termed inflammatory dendritic cells, in inflamed brains from endotoxemic mice. However, we particularly used FcϵRI to characterize these cells, to date probably the best phenotypic marker to distinguish inflammatory dendritic cells from other myeloid cells and avoid giving a mistaken identity (18). Inflammatory dendritic cells characterize for being absent from steady-state tissues and differentiate, as well as microglia (19, 20), from monocytes during inflammation. This would at least partially explain the presence of these cells, as revealed here, in spite of the fact that LPS could actually block the conversion of inflammatory monocytes into dendritic cells *in vivo* (3).

Recent findings support the idea of leukocyte trafficking under physiological and pathological circumstances along circumventricular organs that function as gateways in the CNS such as the choroid plexus (32), a unique neuroimmunological interface positioned to integrate the signals received from the parenchyma with signals coming from circulating immune cells (33–35). Accordingly, we found recruitment but no infiltration of leukocytes in this site along with microglial and astrocytic reactivity after systemic inflammation evoked by LPS as revealed by both the retraction of their processes and enlargement of their cell bodies, which are classic traits of glial activation (5, 16, 36). It should be pointed out that our criteria for the use of the term infiltration was not that given by numerous works as a synonym of recruitment, but the phenomenon by which the cells can invade the parenchyma after their entry through the vessels. This is clearly not only a semantic problem, since the incorrect use of the term may lead to a misunderstanding of the results.

Myeloid cells recruitment to the CNS is a shared characteristic between several autoimmune and neurodegenerative diseases. Chemokine receptors CCR2 and CX_3_CR1 are both essential for myeloid cell trafficking and localization of migrating leukocytes (17, 37) to the perivascular space (2, 38, 39). In this sense and consisting with previous observations (12), we found brain CCL2 production together with CCR2-expressing inflammatory monocytes would ease the selective migration of these cells to the CNS (20, 40–42) following a LPS challenge. Moreover, downregulation of cerebral CX_3_CL1 and its receptor CX_3_CR1 would amplify this response, unleashing microglia pro-inflammatory role by removing the inhibition exerted by CX_3_CL1 (5, 43, 44).

Unlike microglia, LPS priming of isolated inflammatory monocytes led to an induction of T cell proliferative response (45). Despite *ex vivo* re-stimulation with LPS and IFN-g, a potent stimulus for the induction of T cell-suppressive nitric oxide (46, 47), inflammatory monocytes showed responsiveness in the enhancement of T cell proliferation. Zhu et al. (48) clearly showed that CNS isolated inflammatory monocytes could switch their function (49) from antigen presentation to T suppression depending on their activation state and therefore, their ability to produce nitric oxide. In this regard, LPS-treated inflammatory monocytes exhibited an activation threshold higher enough to differentially modulate CD4^+^ (50) and CD8^+^ T cell subsets but not to promote T cell suppression. Besides, the surprisingly functional incompetence of tissue-resident microglia to favor T cell proliferation, which contrasts with other studies done in experimental autoimmune encephalomyelitis (51), highlights the role of peripheral inflammatory monocytes to elicit an appropriate immune response within the CNS in endotoxemic mice.

Type I IFN-mediated recruitment of inflammatory monocytes confirms the importance of these cytokines to orchestrate the trafficking of these cells (52–55) to the CNS (40, 56, 57) and reveals an unknown interaction with CX_3_CL1 to exert its role. Janova et al. (13) recently described an essential function for TLR4 co-receptor CD14 in microglial sensing of CNS damage (6, 10) and further characterized a loop by which IFN-b signaling limits CXCL1 overproduction, thereby modulating myeloid recruitment. Even though CXCL1 appear to be dispensable for neutrophils (14), this could partially explain the reason why these cells still migrate to the CNS albeit type I IFN deficiency.

Although experimental procedures to distinguish properly between microglia and monocytes in CNS impose practical limitations, the results presented here compares the phenotypic and functional features between these tissue-resident vs peripheral recruited cells in LPS-induced neuroinflammation, identifying inflammatory monocytes as key surveillants of the CNS and potential targets for immunotherapy.

## Ethics Statement

All experiments were done in compliance with the procedures outlined in the “Guide for the Care and Use of Laboratory Animals” (NIH Publication No. 86-23, 1985). The experimental protocols were approved by the Institutional Animal Care and Use Committee (IACUC). Our animal facility obtained NIH animal welfare assurance (No. A5802-01, OLAW, NIH, US).

## Author Contributions

JPR conceived and designed the research study, performed the experiments, analyzed data, and wrote the manuscript; CB, EG, and DA performed the experiments and analyzed the data; NB performed the experiments; CRG conceived and designed the research; PI conceived and designed the research study and wrote the manuscript; all authors reviewed the manuscript before submission.

## Conflict of Interest Statement

The authors declare that the research was conducted in the absence of any commercial or financial relationships that could be construed as a potential conflict of interest.

## Supplementary Material

Figure S1 Gating strategy sequence applied for the flow cytometric analysis and cell sorting. Mice were treated i.p. with 40 µg of lipopolysaccharide (LPS) (1,6 mg/kg) for four consecutive days to induce neuroinflammation. Twelve hours post the last injection, mice were euthanized and immune cells were isolated from whole brain homogenates and labeled for subsequent flow cytometric analysis or cell sorting. **(A)** FSC vs SSC, FSC-W vs FSC-H, and SSC-W vs SSC-H flow cytometry density-plots illustrating the gating analysis strategy employed to exclude cell doublets. **(B)** Hierarchical gate view depicting the strategy sequence adopted to assess the phenotypic features of immune cells. Representative rectangle gates were used in all cases for statistical purpose rather than for selection of a cell population.

Figure S2 Two-step intracellular staining Ctrl. Mice were treated i.p. with 40 µg of lipopolysaccharide (LPS) (1,6 mg/kg) for four consecutive days to induce neuroinflammation. Twelve hours post the last injection, mice were euthanized and immune cells isolated and labeled for subsequent flow cytometric analysis. CD11b/Ly6C vs Alexa Fluor 488 flow cytometry density-plots illustrating the secondary antibody control from the intracellular labeling of CCR2 and CX_3_CR1. Representative rectangle gates were used in all cases for statistical purpose rather than for selection of a cell population.

Figure S3 Weight loss comparison between wild-type (WT), IFN-g^−/−^, and IFNAR^−/−^ mice strains following LPS stimulation. Weight loss was assessed in WT, IFN-g^−/−^, and IFNAR^−/−^ mice following the i.p. administration scheme with vehicle or 40 µg of lipopolysaccharide (LPS) (1,6 mg/kg) for four consecutive days to induce neuroinflammation. Results are an average of three independent experiments (*n* = 3–4 animals per group) for each knockout mice strain. Data are expressed as mean ± SEM. Statistical significance levels were set as follows: ^###^ if *p* < 0.001 (WT-Veh, IFN-g^−/−^ Veh, and IFNAR^−/−^ Veh vs WT-LPS, IFN-g^−/−^ LPS, and IFNAR^−/−^ LPS), *** if *p* < 0.001 (WT-LPS vs IFN-g^−/−^ LPS), ^a^ if *p* < 0.001 (WT-LPS vs IFNAR^−/−^ LPS).

Figure S4 Major histocompatibility class II (MHC II) expression comparison between brain-resident CD11b^+^ CD45^lo^ vs peripheral CD11b^+^ CD45^hi^ Ly6C^+^ Ly6G^+^ and CD11b^+^ CD45^hi^ Ly6C^hi^ Ly6G^–^ cells in LPS-treated WT, IFN-g^−/−^, and IFNAR^−/−^ mice strains. Mice were treated i.p. with either vehicle or 40 μg of lipopolysaccharide (LPS) (1,6 mg/kg) for four consecutive days to induce neuroinflammation. Twelve hours post the last injection, mice were euthanised and immune cells were isolated from whole brain homogenates and labelled for subsequent flow cytometric analysis. **(A)** Absolute number and **(B)** mean fluorescence intensity (MFI) of CD11b^+^ CD45^lo^ MHC II^+^ microglial cells, CD11b^+^ CD45^hi^ Ly6C^+^ Ly6G^+^ MHC II^+^ neutrophils and CD11b^+^ CD45^hi^ Ly6C^hi^ Ly6G^–^ MHC II^+^ inflammatory monocytes derived from WT, IFN-g^−/−^ and IFNAR^−/−^ mice, was assessed by flow cytometry. Results are an average of three independent experiments (*n* = 3–4 animals per group) for each knock-out mice strain. Data are expressed as mean ± SEM.
