# Supplementary figures and images for: Type I IFNs Are Required to Promote Central Nervous System Immune Surveillance through the Recruitment of Inflammatory Monocytes upon Systemic Inflammation
Source: Front Immunol. 2017 Dec 4;8:1666. doi: 10.3389/fimmu.2017.01666 (PMC5722985; doi:10.3389/fimmu.2017.01666)

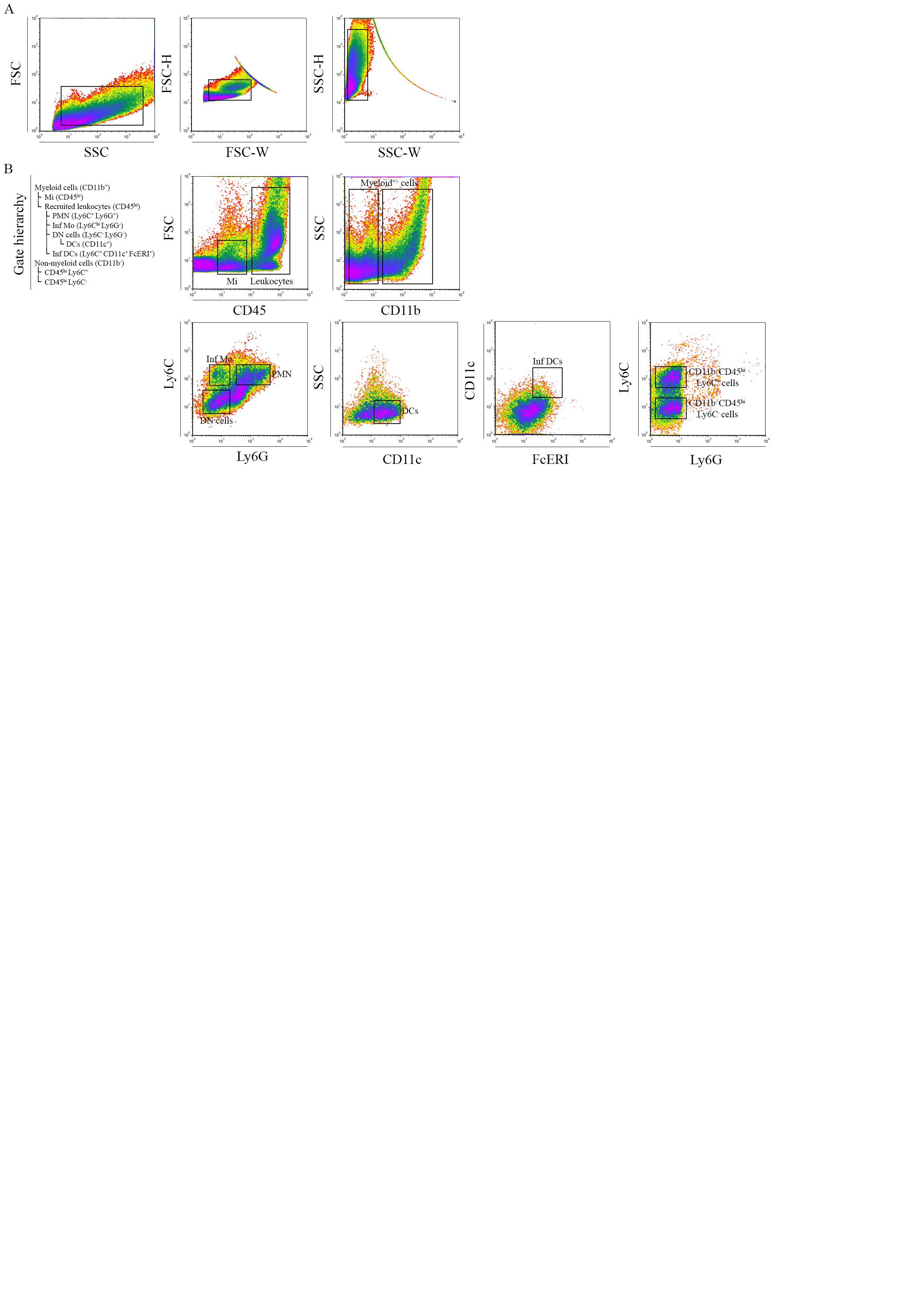

Supplement: Figure S1 — Gating strategy sequence applied for the flow cytometric analysis and cell sorting. Mice were treated i.p. with 40 µg of lipopolysaccharide (LPS) (1,6 mg/kg) for four consecutive days to induce neuroinflammation. Twelve hours post the last injection, mice were euthanized and immune cells were isolated from whole brain homogenates and labeled for subsequent flow cytometric analysis or cell sorting. (A) FSC vs SSC, FSC-W vs FSC-H, and SSC-W vs SSC-H flow cytometry density-plots illustrating the gating analysis strategy employed to exclude cell doublets. (B) Hierarchical gate view depicting the strategy sequence adopted to assess the phenotypic features of immune cells. Representative rectangle gates were used in all cases for statistical purpose rather than for selection of a cell population. [file Image_1.TIF]

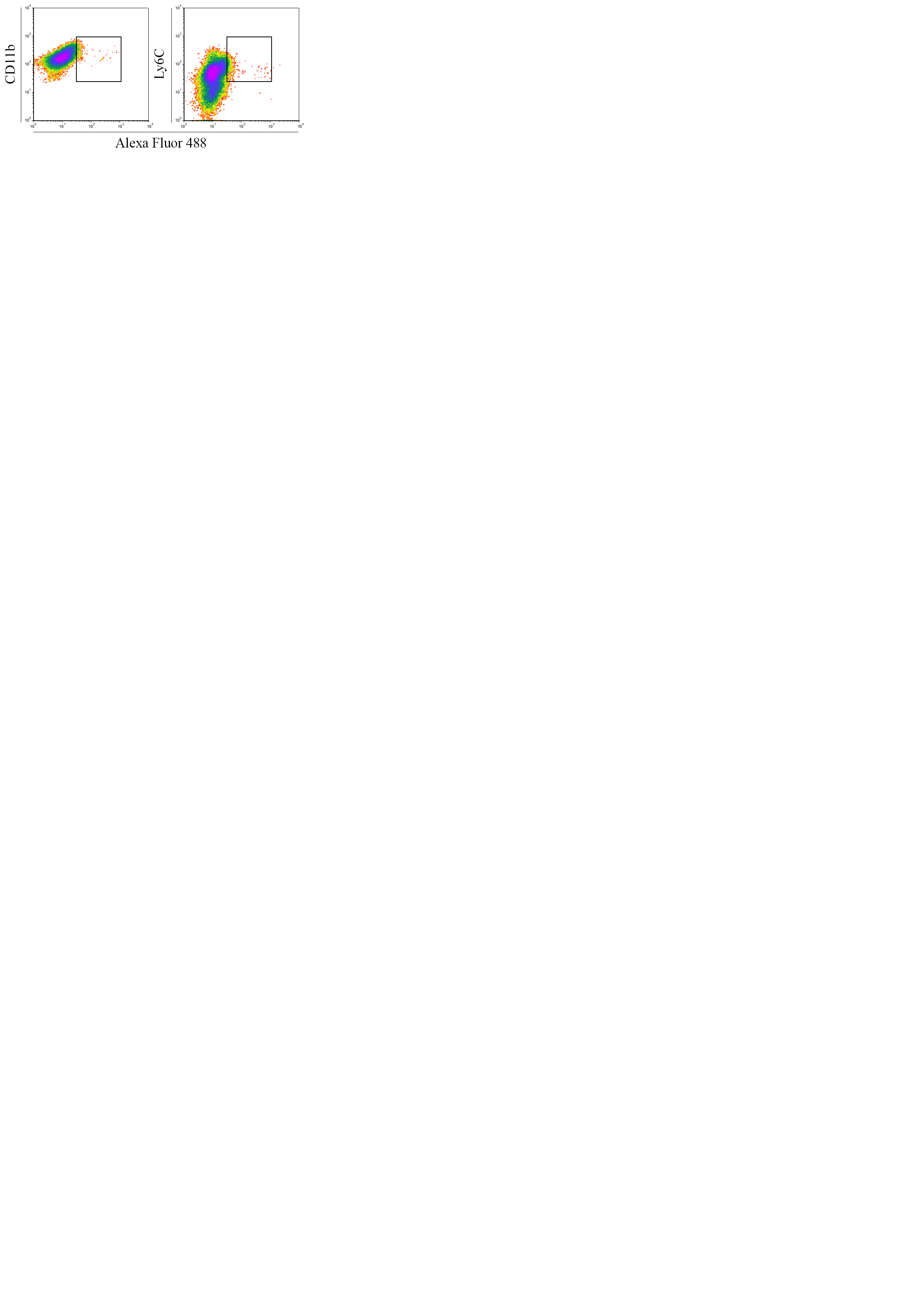

Supplement: Figure S2 — Two-step intracellular staining Ctrl. Mice were treated i.p. with 40 µg of lipopolysaccharide (LPS) (1,6 mg/kg) for four consecutive days to induce neuroinflammation. Twelve hours post the last injection, mice were euthanized and immune cells isolated and labeled for subsequent flow cytometric analysis. CD11b/Ly6C vs Alexa Fluor 488 flow cytometry density-plots illustrating the secondary antibody control from the intracellular labeling of CCR2 and CX3CR1. Representative rectangle gates were used in all cases for statistical purpose rather than for selection of a cell population. [file Image_2.TIF]

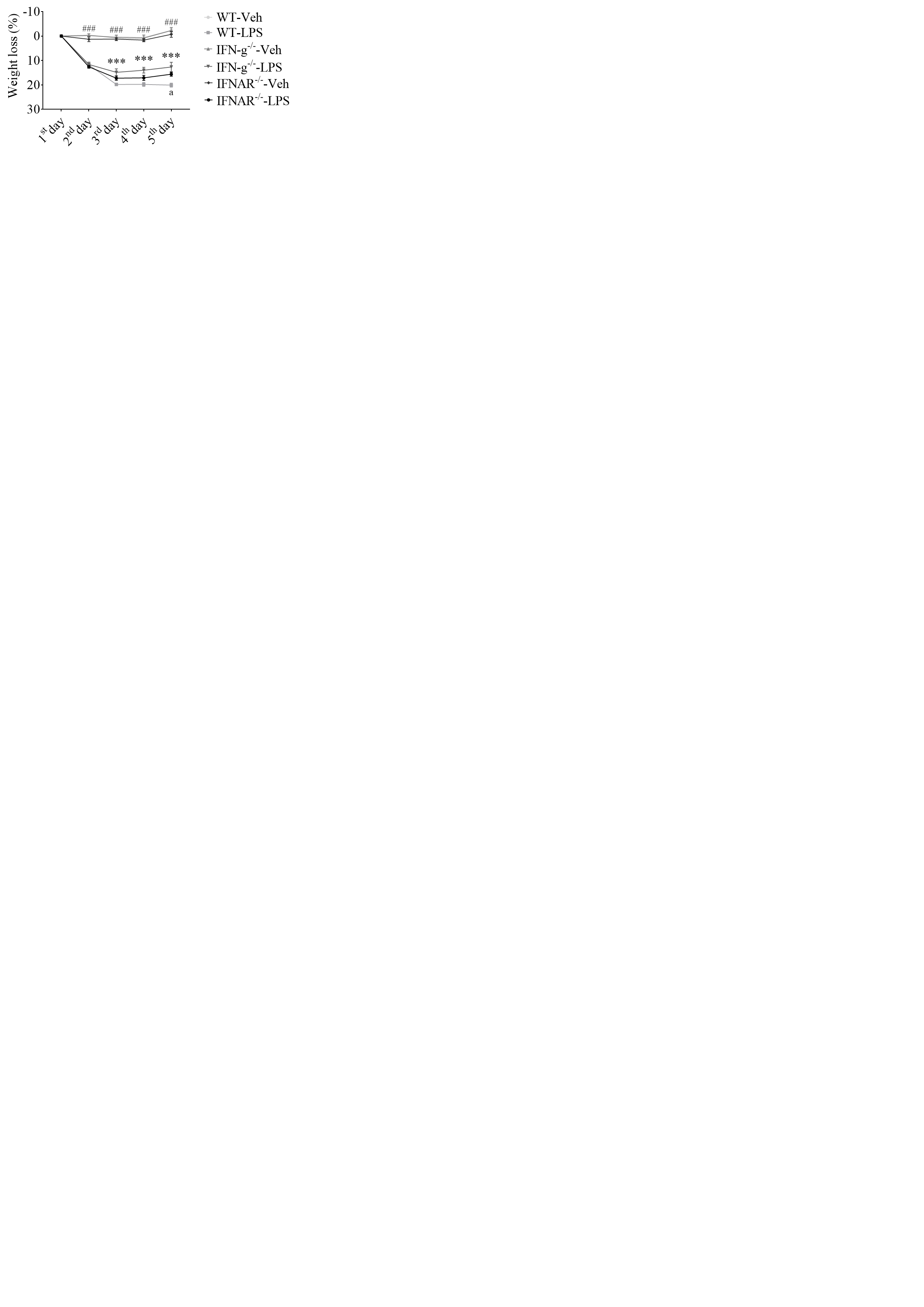

Supplement: Figure S3 — Weight loss comparison between wild-type (WT), IFN-g−/−, and IFNAR−/− mice strains following LPS stimulation. Weight loss was assessed in WT, IFN-g−/−, and IFNAR−/− mice following the i.p. administration scheme with vehicle or 40 µg of lipopolysaccharide (LPS) (1,6 mg/kg) for four consecutive days to induce neuroinflammation. Results are an average of three independent experiments (n = 3–4 animals per group) for each knockout mice strain. Data are expressed as mean ± SEM. Statistical significance levels were set as follows: ### if p < 0.001 (WT-Veh, IFN-g−/− Veh, and IFNAR−/− Veh vs WT-LPS, IFN-g−/− LPS, and IFNAR−/− LPS), *** if p < 0.001 (WT-LPS vs IFN-g−/− LPS), a if p < 0.001 (WT-LPS vs IFNAR−/− LPS). [file Image_3.TIF]

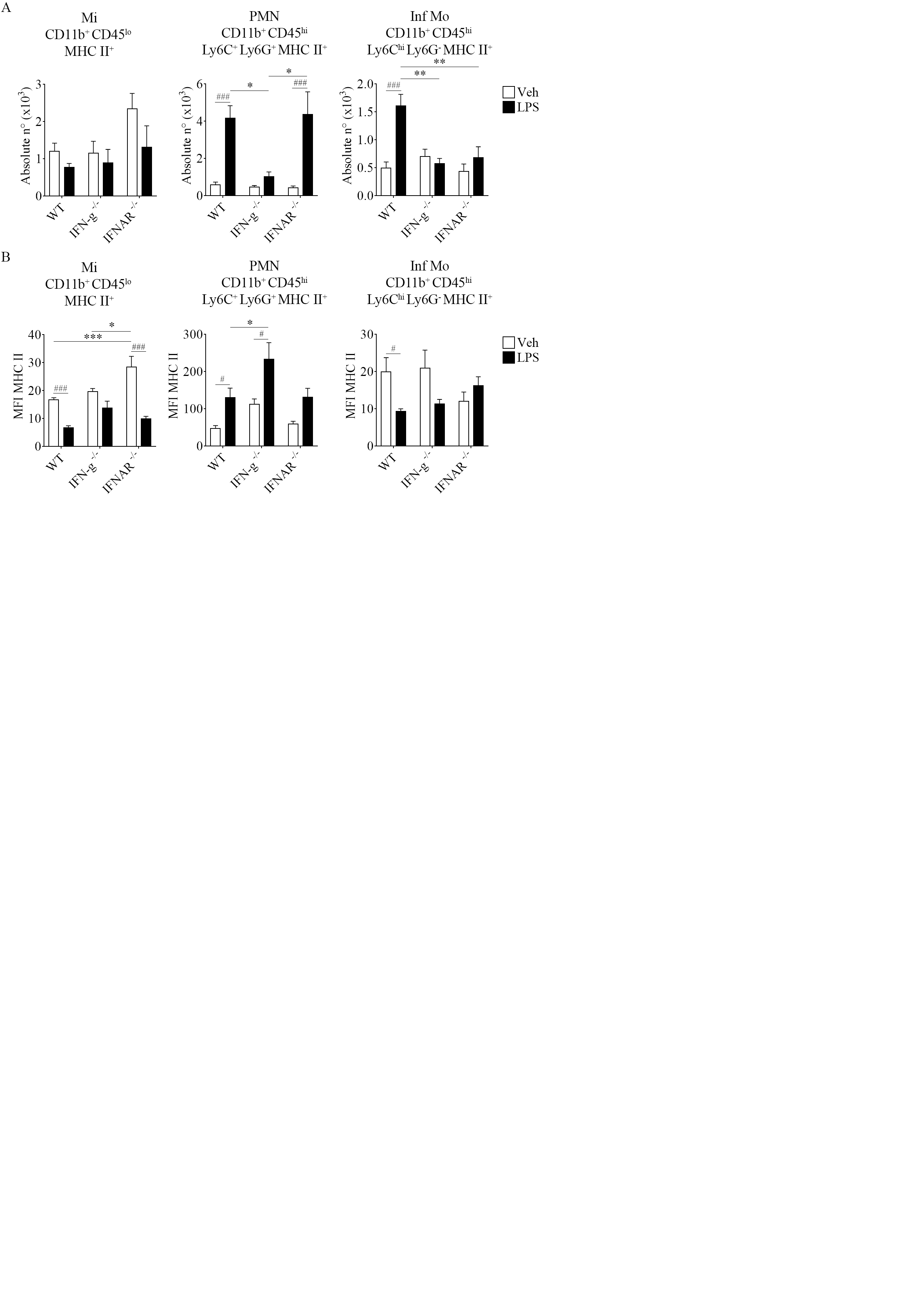

Supplement: Figure S4 — Major histocompatibility class II (MHC II) expression comparison between brain-resident CD11b+ CD45lo vs peripheral CD11b+ CD45hi Ly6C+ Ly6G+ and CD11b+ CD45hi Ly6Chi Ly6G– cells in LPS-treated WT, IFN-g−/−, and IFNAR−/− mice strains. Mice were treated i.p. with either vehicle or 40 μg of lipopolysaccharide (LPS) (1,6 mg/kg) for four consecutive days to induce neuroinflammation. Twelve hours post the last injection, mice were euthanised and immune cells were isolated from whole brain homogenates and labelled for subsequent flow cytometric analysis. (A) Absolute number and (B) mean fluorescence intensity (MFI) of CD11b+ CD45lo MHC II+ microglial cells, CD11b+ CD45hi Ly6C+ Ly6G+ MHC II+ neutrophils and CD11b+ CD45hi Ly6Chi Ly6G– MHC II+ inflammatory monocytes derived from WT, IFN-g−/− and IFNAR−/− mice, was assessed by flow cytometry. Results are an average of three independent experiments (n = 3–4 animals per group) for each knock-out mice strain. Data are expressed as mean ± SEM. [file Image_4.TIF]
